# The association of the pan-immune-inflammation value with mortality in patients monitored in the intensive care unit after cardiopulmonary resuscitation

**DOI:** 10.3389/fmed.2025.1683107

**Published:** 2026-01-08

**Authors:** Muhammed Emin Zora, Ömer Sert

**Affiliations:** 1 Department of Anesthesiology and Reanimation, Faculty of Medicine, Uşak University, Uşak, Türkiye; 2 Anesthesiology and Reanimation Clinic, Uşak Training and Research Hospital, Uşak, Türkiye

**Keywords:** biomarker, cardiopulmonary resuscitation, intensive care unit, mortality, pan-immune-inflammation value, post-cardiac arrest syndrome

## Abstract

**Background:**

Post–cardiac arrest syndrome (PCAS) involves rapid and complex inflammatory responses, yet accessible biomarkers to support early prognostication remain limited. The pan-immune-inflammation value (PIV), derived from routine hematologic indices, has shown prognostic relevance in other inflammatory and malignant conditions; however, its utility in PCAS has not been evaluated.

**Materials and methods:**

This retrospective observational study included 193 adult patients who achieved return of spontaneous circulation following cardiopulmonary resuscitation. Demographic, clinical, and laboratory variables were recorded, along with Sequential Organ Failure Assessment (SOFA) and Acute Physiology and Chronic Health Evaluation II (APACHE II) scores. PIV, Systemic Immune-Inflammation Index (SII), and Systemic Inflammation Response Index (SIRI) values were calculated on day 1 and day 3 of ICU admission. Statistical analyses included Mann–Whitney U, chi-square tests, Spearman correlation, ROC analysis, and multivariable logistic regression.

**Results:**

Of the 193 patients, 85 (44.0%) died within the first 2 days, and 108 (56.0%) survived ≥ 72 h. Non-survivors had significantly higher SOFA and APACHE II scores. PIV was strongly correlated with SII and SIRI; however, neither day 1 nor day 3 PIV values were independently associated with mortality. ROC analysis demonstrated low discriminatory ability for PIV.

**Conclusion:**

Although PIV is an inexpensive and readily accessible marker reflecting systemic inflammation, it did not provide independent prognostic information beyond established clinical severity scores in patients with PCAS. Strong correlations with other leukocyte-based inflammatory indices suggest overlapping biological pathways rather than distinct predictive value. Future multicenter studies with protocolized, more frequent early sampling are needed to characterize early inflammatory responses and identify biomarkers that meaningfully contribute to multimodal prognostication. Until such evidence emerges, PIV should be regarded primarily as a descriptive marker rather than a prognostic tool in this setting.

**Trial registration:**

This single-center retrospective study was approved by the Uşak University Ethics Committee (Decision No: 461-461-08, Date: 07/11/2024).

## Introduction

Cardiopulmonary arrest is a critical medical condition characterized by the sudden and unexpected cessation of cardiac and respiratory functions. This condition often develops due to underlying pathologies such as coronary artery disease (CAD), respiratory failure, and cerebrovascular events (CVE) (1). After cardiopulmonary arrest, post-cardiac arrest syndrome (PCAS) may develop, characterized by brain injury, myocardial dysfunction, a systemic ischemia/reperfusion response, and the continuation of the underlying pathology (2).

Patients who undergo cardiopulmonary resuscitation (CPR) are closely monitored in intensive care units with targeted supportive therapy. These patients are at significant risk of mortality and morbidity; therefore, continuous assessment of their clinical condition and the prevention of potential complications through various biochemical and clinical parameters are of utmost importance (3). Currently, the most commonly used scoring systems in this context are the Sequential Organ Failure Assessment (SOFA) and Acute Physiology and Chronic Health Evaluation (APACHE), which play a crucial role in predicting expected mortality rates (4).

The Pan-Immune-Inflammatory Value (PIV) has emerged as an increasingly studied biomarker in this context. PIV is calculated from neutrophil, platelet, monocyte, and lymphocyte counts and reflects the organism’s overall immune and inflammatory status in a non-specific manner (5). Because PIV is derived entirely from routinely collected hematologic parameters, it requires no additional testing or cost, making its simplicity, low cost, and ease of calculation key advantages when considering it as a potential early prognostic marker.

In a study conducted by Turan, Y. B., It was determined that PIV serves as a prognostic indicator for extended survival in patients experiencing septic shock (6). An elevated PIV indicates heightened inflammation and immune activation, which has been explored as a diagnostic and prognostic biomarker, particularly in various cancers (7, 8).

The Systemic Immune-Inflammation Index (SII) and the Systemic Inflammation Response Index (SIRI) are biomarkers calculated from neutrophil, monocyte, lymphocyte, and platelet counts (9). These biomarkers are derived using a formula similar to PIV. SII has been identified as a prognostic marker in various malignant solid tumors (10). Similarly, SIRI has been reported to possess prognostic significance in pancreatic and nasopharyngeal cancers (11, 12).

To date, no studies have examined the relationship between PIV and mortality in ICU patients after CPR. Our study aims to fill this gap by evaluating the prognostic value of PIV and its association with SII and SIRI. Because PCAS exhibits a sepsis-like inflammatory state with immune dysregulation and multi-organ injury, PIV—an index integrating neutrophil, platelet, monocyte, and lymphocyte counts—may capture this systemic immune-inflammatory response.

## Materials and methods

### Study population

This is a single-center, retrospective observational study conducted in the intensive care unit of a university hospital between July 2022 and July 2024 on patients monitored in the intensive care unit following cardiopulmonary resuscitation for various reasons. A total of 193 patients aged over 18 were included in the study, while patients under 18 were excluded.

The study was approved by the Ethics Committee of Uşak University Faculty of Medicine (Decision No: 461-461-08) and complied with the STROBE (13) guidelines for observational research. The patient’s identity information was kept confidential, all data were anonymized, and access to the hospital database was provided solely for research purposes.

### Data collection

For all patients, the following demographic and clinical data were collected from the hospital’s electronic health records: sex, age, height, weight, SOFA score, APACHE II score, chronic comorbidities, admission diagnosis, CRP, procalcitonin, and lactate levels. The GCS score was defined as the first recorded neurologic assessment upon ICU admission, following return of spontaneous circulation. Additionally, to calculate the PIV, SII, and SIRI values on the first and third days of admission, neutrophil, lymphocyte, monocyte, and platelet counts were retrieved from the hospital system and recorded. For non-PIV laboratory parameters (including lactate, CRP, and procalcitonin), we recorded the first available values obtained within the initial 6 h of ICU admission (day 1) and, for patients who survived ≥ 3 days, repeat measurements obtained within the 24-h period on day 3.

### PIV, SII, and SIRI values

The patients’ values on the first and third days of intensive care unit (ICU) admission were measured, as they may aid in prognostic prediction (14). For patients who experienced mortality, only the values from the first day of admission were recorded. The PIV, SII, and SIRI values were calculated as follows, and their impact on mortality was analyzed:

PIV = [neutrophil count (10^3^/μL) × platelet count (10^3^/μL) × monocyte count (10^3^/μL)]/lymphocyte count (10^3^/μL)

SII = neutrophil count (10^3^/μL) × platelet count (10^3^/μL)/lymphocyte count (10^3^/μL)

SIRI = neutrophil count (10^3^/μL) × monocyte count (10^3^/μL)/lymphocyte count (10^3^/μL)

### Statistical analysis

Statistical analyses were performed using the IBM SPSS Statistics 26 software package. Continuous variables’ normality was assessed using visual methods (histograms and probability plots) and analytical methods (Kolmogorov-Smirnov test). Continuous variables were compared using the Student’s *t*-test or the Mann-Whitney U test, while categorical variables were analyzed using the chi-square test. Relationships between variables, at least one of which did not follow a normal distribution, were evaluated using Spearman’s correlation test.

The diagnostic performance of the Pan-Immune-Inflammation Value (PIV) values on the first day (PIV_1) and the third day (PIV_3) in predicting mortality was assessed using Receiver Operating Characteristics (ROC) analysis. The area under the curve (AUC), sensitivity, and specificity values were calculated. Additionally, multivariable logistic regression analysis was conducted to evaluate the impact of independent variables on mortality. The difference between PIV_1 and PIV_3 values was analyzed using the Wilcoxon Signed-Rank Test.

Variables with *p* < 0.05 in the univariate analysis (SOFA score, APACHE II score, age, PIV, SII, and SIRI) were entered into the multivariate logistic regression model. These three biomarker indices were strongly correlated; therefore, they were included together to evaluate whether any retained an independent effect. None of them remained significant once included in the same model.

Because PIV, SII, and SIRI are derived from overlapping leukocyte- and platelet-based components, collinearity among these indices was expected. Their simultaneous inclusion in multivariable regression was performed as an exploratory analysis and may limit the ability to determine independent effects. Therefore, the multivariate modeling should be interpreted as hypothesis-generating rather than as a definitive assessment of prognostic performance. Future studies may benefit from alternative model structures—such as separate models for each biomarker or dimensionality-reduction approaches—to evaluate independent associations better.

This study did not aim to develop or validate a combined prognostic scoring model; instead, the analyses focused on evaluating whether each biomarker demonstrated independent associations with mortality.

A *p* < 0.05 was considered statistically significant in all statistical analyses.

## Results

### Baseline characteristics

**FIGURE 1:**
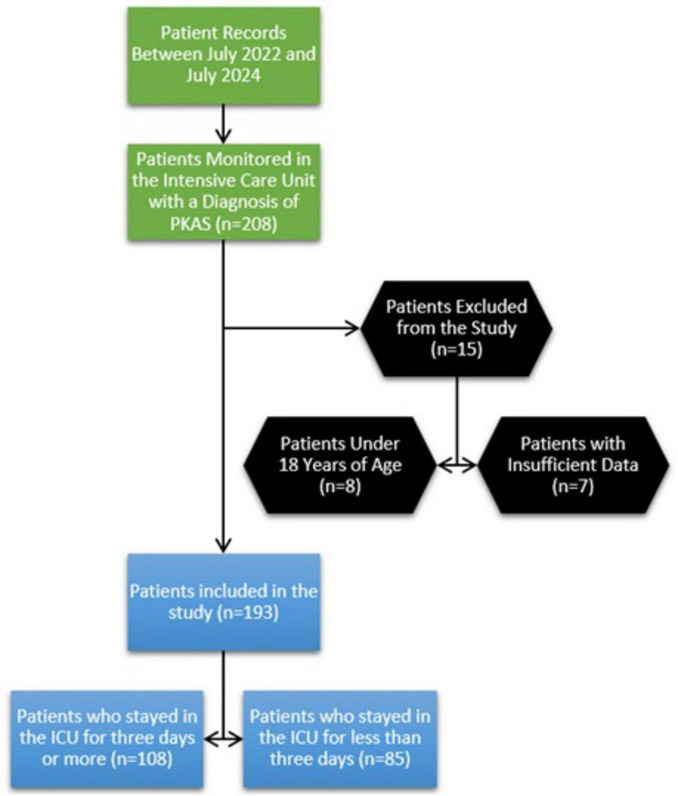
Flowchart of the patients included in the study.

Patients diagnosed with PCAS and monitored in the intensive care unit were identified from hospital information system records between July 2022 and July 2024 (*n* = 208). Eight patients were under 18, and seven were excluded due to insufficient data. As a result, the study encompassed 193 patients. Of these, 85 individuals (44.04%) underwent intensive care for fewer than 3 days, whereas 108 patients (55.96%) required intensive care for 3 days or longer. The flowchart of the study patients is presented in Figure 1.

The median age of the patients was 69.0 years, and no significant sex-based difference in mortality was observed (*p* = 0.624) (Table 1). SOFA and APACHE II scores were significantly lower in surviving patients compared with non-survivors (*p* < 0.001). Survivors also demonstrated longer ICU stays and higher GCS scores (*p* < 0.001). Comorbidities showed no significant differences in distribution between survivors and non-survivors; detailed frequencies are presented in Table 1.

**TABLE 1:** Baseline characteristics of the study cohort and association with mortality.

Characteristics	All patients (*N* = 193)	Survivor (*N* = 32)	Non-survivor (*N* = 161)	*P*-value^a^
Gender (F/M) (%)	86/107 (44.3/55.2)	13/19 (15.1/17.8)	73/88 (84.9/82.2)	0.624*
Age (year) [median (IQR)]	69.0 (60.0, 79.5)	67.0 (59.5, 73.0)	69.0 (60.0, 81.0)	**0.034** **
ICU time (day) [median (IQR)]	3 (1.0, 7.0)	5.5 (3.0, 13.75)	2 (1.0, 7.0)	**<0.001^#^**
GCS [median (IQR)]	3 (3.0, 3.0)	5 (3.0, 7.0)	3 (3.0, 3.0)	**<0.001^#^**
SOFA [median (IQR)]	13 (11.0, 17.0)	8.5 (7.0, 10.75)	14 (12.0, 17.75)	**<0.001^#^**
APACHE [median (IQR)]	33 (27.0, 38.0)	19 (15.0, 26.5)	34 (30.0, 39.0)	**<0.001^#^**
Coronary artery disease (n) (%)	103 (53.4)	14 (13.6)	89 (55.3)	0.250
Chronic obstructive pulmonary disease (n) (%)	38 (19.7)	7 (18.4)	31 (81.6)	0.808
Diabetes mellitus (n) (%)	38 (19.7)	7 (18.4)	31 (81.6)	0.808
Hypertension (n) (%)	30 (15.5)	6 (20)	24 (80)	0.596
Renal failure (n) (%)	21 (10.9)	4 (19)	17 (81)	0.756
Stroke (n) (%)	13 (6.7)	0	13 (100)	0.096
Pneumonia (n) (%)	12 (6.2)	2(16.7)	10 (83.3)	0.993
Solid malignant tumors (n) (%)	10 (5.2)	1 (10)	9(90)	0.566
Other (n) (%)	21 (10.9)	4 (19)	17 (81)	0.747

^a^The *P*-values for the comparison of survivor and non-survivor groups, *Chi-Square, **Student’s *t*-test, #Mann-Whitney U. Bold values indicate statistical significance (*p* < 0.05).

The biochemical and hematologic values of patients on Days 1 and 3 are summarized in Table 2. Surviving patients had significantly lower procalcitonin, lactate, and Day-3 CRP levels, as well as higher platelet counts on day 1 (all *p* < 0.05). Other routine hematologic parameters showed no clinically meaningful differences between survivors and non-survivors.

**TABLE 2:** Comparison of biomarkers and hemogram values.

Variables	All patients (*N* = 193)	Survivor (*N* = 32)	Non-survivor (*N* = 161)	*P*-value^#^
Neutrophil_1	10.23 (6.54, 17.33)	10.13 (8.56, 15.18)	10.37 (5.85, 17.73)	0.880
Lymphocyte_1	1.65 (0.79, 4.71)	1.68 (0.73, 3.85)	1.65 (0.79, 4.81)	0.436
Monocyte_1	0.57 (0.33, 0.79)	0.57 (0.40, 0.79)	0.57 (0.33, 0.79)	0.800
Platelet_1	209 (154, 289)	242 (197, 327)	204 (148, 285)	**0.039**
CRP_1	44.3 (10.9, 93.4)	27.6 (4.7, 87.3)	46.2 (0.69, 94.4)	0.106
Procalcitonin_1	2.13 (0.59, 6.23)	0.82 (0.43, 2.25)	2.87 (0.69, 8.65)	**0.004**
Lactate_1	7.0 (2.4, 12.8)	3.1 (1.9, 5.5)	8.4 (2.6, 13.5)	**<0.001**
PIV_1	755.98 (169.75, 1851.46)	687.58 (486.34, 1954.08)	762.99 (141.91, 1851.46)	0.327
SII_1	1408.05 (454.21, 3571.04)	1762.64 (803.00, 3665.47)	1346.54 (326.96, 3487.65)	0.180
SIRI_1	3.43 (0.85, 9.44)	4.68 (1.36, 10.54)	2.99 (7.28, 9.44)	0.444
Neutrophil_3	9.89 (7.12, 14.29)	8.28 (6.20, 12.71)	10.39 (7.33, 14.78)	0.053
Lymphocyte_3	0.82 (0.59, 1.29)	0.79 (0.53, 1.13)	0.96 (0.62, 1.42)	0.199
Monocyte_3	0.52 (0.31, 0.82)	0.46 (0.31, 0.73)	0.53 (0.30, 0.86)	0.414
Platelet_3	182 (109, 239)	193 (141, 263)	172 (97, 236)	0.073
CRP_3	77.8 (38.1, 149.2)	36.5 (12.8, 69.5)	95.4 (54.0, 157.7)	**<0.001**
Procalcitonin_3	2.07 (0.798, 10.68)	0.99 (0.25, 2.98)	2.32 (0.98, 18.51)	**0.002**
Lactate_3	2.1 (1.5, 3.5)	1.5 (1.1, 1.9)	4.5 (1.7, 4.5)	**<0.001**
PIV_3	1002.28 (441.93, 2102.98)	1012.89 (475.89, 2205.08)	992.89 (410.81, 1973.92)	0.812
SII_3	1826.78 (954.14, 3506.41)	2539.75 (1188.60, 3663.27)	1604.10 (907.52, 3374.48)	0.192
SIRI_3	6.50 (3.09, 10.04)	6.31 (2.93, 8.60)	6.57 (3.09, 11.37)	0.408

#Mann-Whitney U, data are expressed as median (IQR). Neutrophil_1, Neutrophil on day 1; Lymphocyte_1, Lymphocyte on day 1; Monocyte_1, Monocyte on day 1; Platelet_1, Platelet on day 1; CRP_1, CRP on day 1; Procalcitonin_1, Procalcitonin on day 1; Lactate_1, Lactate on day 1; PIV_1, PIV on day 1; SII_1, SII on day 1; SIRI_1, SIRI on day 1; Neutrophil_3, Neutrophil on day 3; Lymphocyte_3, Lymphocyte on day 3; Monocyte_3, Monocyte on day 3; Platelet_3, Platelet on day 3; CRP_3, CRP on day 3; Procalcitonin_3, Procalcitonin on day 3; Lactate_3, Lactate on day 3; PIV_3, PIV on day 3; SII_3, SII on day 3; SIRI_3, SIRI on day 3. Day 3 values include only patients surviving ≥ 72 h; comparisons may reflect survivor bias. Bold values indicate statistical significance (*p* < 0.05).

Day-1 and Day-3 biomarker values (PIV, SII, and SIRI) are presented in Table 2; although numerical trends were observed, none demonstrated significant prognostic value, and Day-1 vs. Day-3 comparisons are subject to survival bias.

Strong and positive correlations were observed between PIV_1 and SIRI_1 (*r* = 0.964, *p* < 0.01) as well as between PIV_1 and SII_1 (*r* = 0.906, *p* < 0.01). A similar relationship was also identified between SIRI_1 and SII_1 (*r* = 0.891, *p* < 0.01). All correlations were statistically significant (*p* < 0.01).

On the third day, the PIV_3, SII_3, and SIRI_3 correlations were analyzed using Spearman’s rho method. A strong positive correlation was identified between PIV_3 and SII_3 (*r* = 0.790, *p* < 0.01) as well as between PIV_3 and SIRI_3 (*r* = 0.855, *p* < 0.01). A moderate positive correlation was also observed between SII_3 and SIRI_3 (*r* = 0.618, *p* < 0.01). All correlations were statistically significant (*p* < 0.01).

According to the regression analysis results, the effects of the variables PIV_1 (*R* = 0.001, *p* = 0.629) and SIRI_1 (*R* = 0.002, *p* = 0.517) on mortality were found to be minimal and statistically insignificant, whereas SII_1 (*R* = 0.000, *p* = 0.776) was determined to have no effect.

**FIGURE 2:**
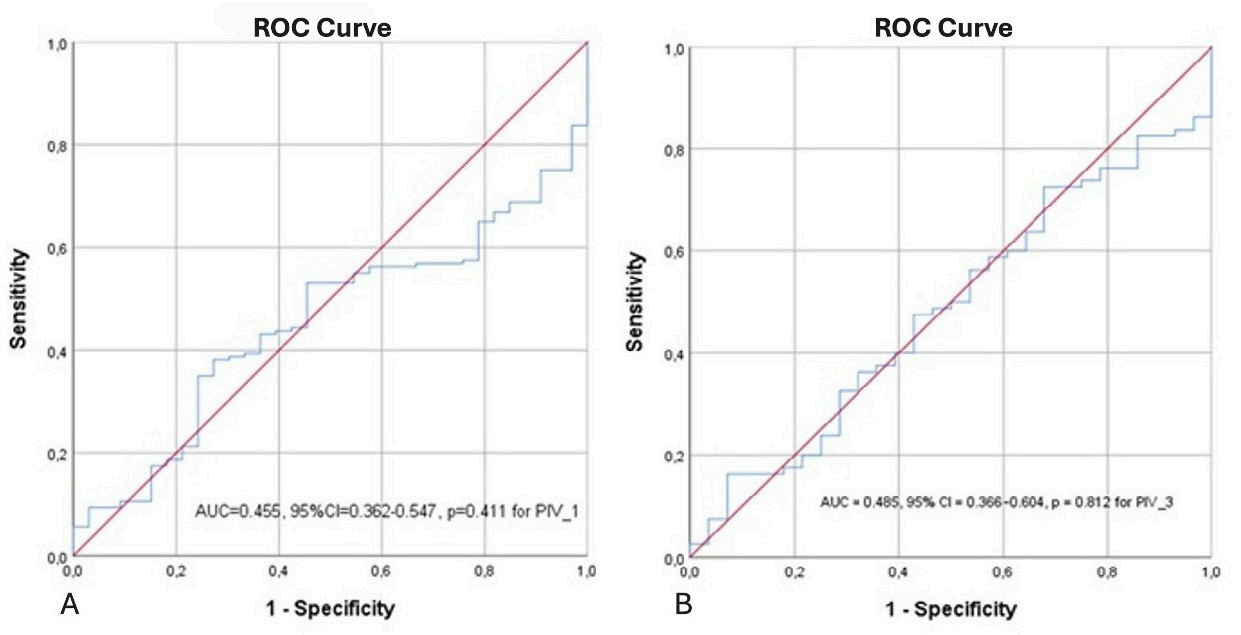
ROC analysis demonstrating the relationship between PIV_1 and mortality **(A)** and PIV_3 and mortality **(B)**.

The ROC curve illustrating the predictive value of PIV_1 for mortality is presented in Figure 2A. (AUC = 0.455, 95% CI = 0.362–0.547, *p* = 0.411 for PIV_1) (Figure 2A). This AUC indicates no discriminatory ability of PIV_1 for predicting mortality.

According to the results of the ROC analysis, the predictive power of the PIV_3 variable for mortality was found to be weak (AUC = 0.485, 95% CI: 0.366–0.604, *p* = 0.812) (Figure 2B), confirming that PIV_3 had no discriminatory ability.

### Multivariate logistic regression

Variables with *p*-values less than 0.05 in the univariate analysis were entered into the multivariate logistic regression model. As presented in Table 3, none of the biomarker indices (PIV, SII, SIRI) were independently associated with early mortality. Among the variables included in the model, only the APACHE II score remained a significant predictor of early mortality (OR 1.26; 95% CI 1.09–1.46; *p* = 0.001) (Table 3). Covariates included SOFA score, APACHE II score, age, and the three biomarker indices (PIV, SII, SIRI). Although the three biomarkers were strongly correlated, they were included together; none showed an independent association.

**TABLE 3:** Multivariate logistic regression analysis for predictors of early mortality.

Variable	OR[Exp(B)]	95% CI for OR	*p*-value
PIV (day 1)	1.000	1.000–1.000	0.192
SII (day 1)	1.000	1.000–1.000	0.575
SIRI (day 1)	1.148	0.947–1.393	0.161
SOFA score	1.038	0.754–1.429	0.819
APACHE II score	1.263	1.094–1.458	0.001
Age	1.003	0.967–1.040	0.870

OR, odds ratio; CI, confidence interval.

In a secondary analysis limited to patients who survived at least 3 days, we built an additional multivariate model using Day 3 biomarker values (Table 4). These results were consistent with the main model: none of the biomarkers (PIV, SII, SIRI) were independently associated with early mortality, while APACHE II remained significant.

**TABLE 4:** Multivariate logistic regression analysis using Day 3 biomarker values.

Variable	OR [Exp(B)]	95% CI for OR	*p*-value
SOFA score	1.110	0.762–1.618	0.585
APACHE II score	1.198	1.027–1.396	0.021
Age	0.999	0.957–1.042	0.950
PIV (day 3)	1.000	0.999–1.001	0.625
SII (day 3)	1.000	0.999–1.000	0.272
SIRI (day 3)	1.125	0.936–1.352	0.210

OR, odds ratio; CI, confidence interval. Multivariate analysis includes only patients surviving ≥ 72 h. Model reflects an exploratory evaluation; overlapping hematologic components of PIV, SII, and SIRI may introduce multicollinearity and limit assessment of independent effects.

## Discussion

PCAS patients in intensive care units represent a clinically complex group in whom early prognostication remains challenging. Following return of spontaneous circulation, patients undergo multifactorial injury processes, including global ischemia–reperfusion injury, myocardial dysfunction, and neurological impairment, in addition to the initial etiology of arrest (1). Given these overlapping mechanisms and their rapid evolution in the early post-arrest period, identifying a single inflammatory biomarker with consistent prognostic value is inherently tricky. Interest has therefore grown in simple and readily available biomarkers—such as the inexpensive, routinely obtained PIV—which have shown prognostic relevance in other clinical settings; however, in our cohort, it did not demonstrate an independent association with early mortality after adjustment for established clinical predictors, indicating that leukocyte-based indices may offer limited incremental utility in this population.

Several factors may account for the lack of an independent association between PIV and mortality in this cohort. The inflammatory response following return of spontaneous circulation is highly dynamic, with cytokine levels peaking within hours and declining rapidly thereafter; therefore, measurements obtained only on day 1 and day 3 may not have captured the most informative early window of immune activation. Because these rapid cytokine-driven changes often precede measurable shifts in leukocyte and platelet counts, PIV may fail to reflect the earliest and most prognostically relevant phase of immune dysregulation. In addition, although PIV has demonstrated utility in conditions characterized by chronic or sustained inflammation, it may be less sensitive to the abrupt, biphasic immunologic shifts that characterize post–cardiac arrest syndrome, where early endothelial and cytokine-mediated processes may occur before detectable changes in hematologic indices. Furthermore, heterogeneity in arrest etiologies and pre-arrest physiology likely contributed to substantial variability in inflammatory trajectories, reducing the ability to detect consistent biomarker–outcome associations.

Despite advances in post–cardiac arrest care, mortality rates remain high. Adrie et al. first described a “sepsis-like” syndrome after cardiac arrest, characterized by increased inflammatory mediators and endotoxemia, suggesting a mechanistic overlap with critical illness–related systemic inflammation (15, 16). Subsequent studies have reported associations between cytokines such as IL-6 and mortality, though findings have varied across cohorts (17–19). These observations collectively highlight the intensity and complexity of post-resuscitation immune activation; however, how these cytokine-driven pathways translate into changes in composite hematologic indices such as PIV remains uncertain. PCAS involves a biphasic immune response—early endothelial injury, complement activation, and cytokine release followed by immune suppression—a pattern not fully captured by leukocyte- and platelet-derived indices. Accordingly, although PIV may reflect systemic inflammation, it appears insufficient to distinguish outcomes in a condition in which organ dysfunction, endothelial injury, and non-hematologic molecular processes exert a more substantial prognostic influence.

PIV is a composite biomarker derived from four peripheral blood cell parameters and was developed to reflect systemic inflammatory activation (20). Initially described as a prognostic marker in metastatic colorectal cancer (21), elevated PIV has since been linked to adverse outcomes in several malignancies (6, 22) and in non-oncologic inflammatory conditions such as acute heart failure (23) and ANCA-associated vasculitis (24). In these settings, PIV appears to function as a marker of sustained or chronically amplified inflammation, in which hematologic shifts evolve over more extended periods and are more closely aligned with the underlying disease trajectory. These findings suggest that PIV reflects sustained inflammatory activity; however, in our post–cardiac arrest cohort, PIV was not associated with mortality and did not offer incremental prognostic value beyond established predictors. This contrast likely reflects the fundamentally different immunopathology of PCAS, where early cytokine-mediated and endothelial processes occur rapidly and may resolve or progress before changes in leukocyte- and platelet-based indices become detectable.

Peberdy et al. reported that inflammatory markers peak early after cardiac arrest and remain elevated for up to 24 h without further increases in non-survivors (17). Subsequent studies have emphasized the need for more frequent sampling during CPR and immediately after ROSC to characterize the rapid post-arrest inflammatory trajectory (25–27). These findings suggest that the most prognostically informative immunologic changes may occur within a narrow early window that is not captured by routine clinical sampling. Consistent with this literature, we assessed biomarkers on day 1 and day 3; however, no meaningful temporal changes were observed, and neither early nor later PIV measurements were associated with mortality. Importantly, Day-3 measurements were available only for patients who survived at least 72 h, introducing survivor bias that limits the interpretation of temporal trends. Together, these issues indicate that the temporal resolution of leukocyte- and platelet-derived indices, such as PIV, may be insufficient to track the rapid, transient inflammatory fluctuations characteristic of PCAS.

Several scoring systems are used in intensive care settings to assess disease severity and estimate prognosis. Although each has limitations, the APACHE II score remains widely used for mortality prediction (28), while the SOFA score is applied to quantify organ dysfunction (29). Consistent with prior studies, our cohort exhibited elevated APACHE II and SOFA scores, reflecting advanced age, comorbidities, and illness severity. In multivariable analysis (Table 3), these clinical indices remained independently associated with mortality, whereas PIV offered no additional prognostic value. This finding suggests that when robust, multi-parameter clinical scoring systems already account for much of the variation in patient outcomes, leukocyte-based inflammatory indices may contribute little incremental information. Accordingly, the prognostic utility of PIV in PCAS appears constrained by both its biological limitations and the strong predictive capacity of established severity scores.

Sepsis and septic shock remain major global health concerns associated with substantial morbidity and mortality (30). Given the immunologic overlap between PCAS and sepsis (15), we measured CRP and procalcitonin, along with hematologic indices. In our cohort, procalcitonin levels were significantly higher in non-survivors on both day 1 and day 3, whereas CRP increased significantly only on day 3. These findings are consistent with the concept that cytokine-related biomarkers, which respond rapidly to early inflammatory surges, may better reflect the acute immunologic disturbances characteristic of PCAS. By contrast, PIV—derived from leukocyte and platelet distributions—showed no association with mortality, suggesting that cellular indices may respond more slowly or less specifically to the abrupt, cytokine-driven processes occurring after cardiac arrest. Accordingly, traditional inflammatory markers such as CRP and procalcitonin appear to retain greater prognostic relevance in the early post-resuscitation period than composite hematologic indices, such as PIV.

SII and SIRI are composite inflammatory indices incorporating neutrophil, monocyte, platelet, and lymphocyte counts, similar to PIV (31). Although these markers have been evaluated in various disease states, their prognostic significance in post–cardiac arrest syndrome is not well defined. In our cohort, PIV showed strong positive correlations with both SII and SIRI, indicating substantial conceptual and hematologic overlap. Such overlap naturally introduces multicollinearity in multivariable analyses, reducing the ability to distinguish independent associations and limiting the apparent prognostic contribution of any single biomarker. Consistent with this, none of the three indices demonstrated an independent association with mortality, suggesting that their shared cellular components reflect similar inflammatory information rather than distinct prognostic signals. In addition, factors such as early neurological status and family decisions regarding goals of care often play a substantial role in post–cardiac arrest outcomes, which may lessen the observable impact of leukocyte- or platelet-derived indices. Thus, both biological overlap among these indices and the influence of non-inflammatory determinants of outcome likely contribute to the limited incremental prognostic value of PIV in this population.

Overall, our findings suggest that while PIV correlates with other leukocyte-based inflammatory indices, it does not provide prognostic information beyond established severity scores in post–cardiac arrest syndrome. This pattern indicates that hematologic indices derived from neutrophil, monocyte, lymphocyte, and platelet counts may not adequately capture the rapid, cytokine-driven immune dysregulation characteristic of the immediate post-resuscitation period. In PCAS, early endothelial injury, complement activation, and molecular inflammatory cascades often precede or evolve independently of measurable shifts in peripheral blood cell distributions, limiting the ability of composite hematologic markers to reflect clinically meaningful differences in disease trajectory. Accordingly, PIV may be better interpreted as a descriptive marker of inflammation rather than a standalone prognostic tool in this setting. Prospective studies with earlier and more frequent sampling are therefore needed to characterize these fast-evolving immunologic processes and to identify biomarkers that more accurately reflect the underlying pathophysiology of PCAS and have the potential to contribute meaningfully to multimodal prognostication.

### Study limitations

This study has several limitations. First, it was conducted retrospectively at a single center, which limits generalizability and precludes establishing causal relationships. The heterogeneous etiologies of cardiac arrest in the study population may also have affected clinical homogeneity and contributed to variability in biomarker responses. In addition, the sample size, particularly in subgroup analyses, may limit statistical power; therefore, effect sizes should be interpreted cautiously and confirmed in larger multicenter cohorts.

Second, although we evaluated inflammatory biomarkers at two time points, this approach may not fully capture the rapid immunological fluctuations occurring within the first hours after return of spontaneous circulation. Biomarker sampling was performed according to routine clinical practice rather than a protocolized timeline, and early cytokine surges may not have been measured. Thus, the temporal behavior of inflammatory markers in the immediate post-arrest period remains incompletely characterized in this cohort.

Third, day-3 biomarker values were available only for patients who survived at least 72 h; thus, longitudinal comparisons reflect a survivor-enriched subgroup rather than the entire cohort. This survival bias may obscure associations present in patients with early mortality and limit the interpretability of temporal patterns.

Fourth, PIV, SII, and SIRI are mathematically correlated indices based on overlapping leukocyte and platelet parameters. Their simultaneous inclusion in multivariable models may introduce multicollinearity, reducing the ability to assess independent effects and contributing to model instability. Accordingly, these analyses should be viewed as exploratory, and future studies may benefit from independent modeling or dimension-reduction techniques to clarify the distinct contributions of individual indices.

Fifth, although PIV demonstrated low discriminatory performance, additional predictive performance metrics, such as calibration statistics, net reclassification improvement, or external validation, were not reported. Therefore, the prognostic implications of PIV should be interpreted as hypothesis-generating rather than definitive.

Finally, while PIV is simple and inexpensive to obtain, it has not been validated as a prognostic tool in PCAS. Because PIV reflects peripheral leukocyte and platelet distribution rather than cytokine levels of activation, it may fail to capture early endothelial and molecular immune responses characteristic of ischemia–reperfusion injury (15, 17, 29). This biological discordance likely contributes to the lack of association observed in this cohort but does not preclude further investigation of improved or complementary biomarkers.

These limitations collectively highlight the need for larger, prospective multicenter studies with protocolized early sampling and refined analytical strategies before PIV can be considered for routine clinical application.

## Conclusion

Our findings indicate that although PIV is a simple, inexpensive, and readily available biomarker, it was not independently associated with mortality in post–cardiac arrest syndrome and did not add prognostic value beyond established severity scores. While PIV correlated strongly with other leukocyte-based inflammatory indices, this overlap did not confer meaningful discriminatory ability, suggesting that peripheral blood cell–derived markers may not capture the early cytokine-mediated immune responses characteristic of ischemia–reperfusion injury.

These results highlight the need for prospective, multicenter studies with protocolized, more frequent early sampling to better characterize the temporal evolution of inflammation after cardiac arrest and to identify biomarkers that may contribute to a multimodal prognostic approach. Until such evidence becomes available, PIV should be interpreted primarily as a descriptive measure of systemic inflammation rather than a standalone prognostic tool in this population. While earlier and more frequent sampling may help clarify inflammatory dynamics, future research should prioritize identifying alternative biomarkers or mechanisms that more directly reflect the underlying pathophysiology of PCAS, rather than repeating the same approach with larger samples.

## Data Availability

The raw data supporting the conclusions of this article will be made available by the authors, without undue reservation.
